# Sweetness preference and its impact on energy intake and body weight – a review of evidence

**DOI:** 10.3389/fnut.2023.1289028

**Published:** 2023-10-18

**Authors:** Philip Prinz

**Affiliations:** Department of Nutritional Sciences, German Sugar Association, Berlin, Germany

**Keywords:** dietary sweetness, sweetness preference, energy intake, body weight, reformulation

## Abstract

In the last few years, several approaches have been postulated for tackling the global increase in overweight and obesity rates, including different dietary macronutrient compositions or the timing of meals. Recently, taste modulation has come into focus as a possible approach for influencing dietary behavior. The perception of sweet taste is innate and an evolutionary protection mechanism to prevent primates from eating poisonous plants. It is hypothesized that this innate sweetness preference could be modulated by dietary sweetness, including sweet foods and beverages, which results in a learned sweetness preference that affects energy intake and body weight. However, this hypothesis is not supported by unanimous scientific evidence. This review provides an update of the current literature, regarding the modulation of sweetness preference as a possible new approach in the prevention of overweight and obesity. In general, results from observation as well as interventional studies in all age groups are heterogeneous. The majority showed no effect of dietary sweetness modulation on sweetness preference, energy intake or anthropometric measures. Therefore, the modulation of sweetness preference due to diet, foods or beverages is still a hypothesis and not scientifically proven. However, due to the lack of data, more research is necessary.

## 1. Introduction

The preference for sweetness is universal in primates (humans and monkeys) and can be distinguished in two major ways (1, 2). First, the ‘innate sweetness preference’, which is an evolutionarily driven taste preference, characterized by preference for higher levels of sweetness during infancy and childhood (3, 4). Sweetness preference peaks in childhood and adolescence before declining again with increasing age (1). All primates prefer sweet tastes and reject other tastes, such as bitter ones (2). This can be derived from the fact that, especially in the plant world, if a sweet taste predominates, the plant in question will be considered to be safe for the body and will be ingested (5). Second, the ‘learned sweetness preference,’ which is a consequence of modulated dietary sweetness or modulated exposure to sweet tasting foods and beverages. For example, it is hypothesized that increased sustained dietary sweetness may promote a generalized desire for sweet foods and beverages, i.e., the development of a “sweet tooth” (3, 4). A “sweet tooth” may trigger excess energy intake due to higher intakes of palatable and energy-dense foods and, consequently, body weight gain (6, 7).

Naturally occurring dietary sugars, among others, are fructose, sucrose, and glucose, which are sources of both energy and sweetness (1). Today, the discussion of sugar consumption is often the main focus in connection with the development of overweight and obesity as well as non-communicable diseases (NCDs), including type 2 diabetes mellitus or cardiovascular diseases. In particular, sugar-sweetened beverages (SSBs) are linked to diverse health issues, including overweight and obesity or type 2 diabetes mellitus in prospective observational studies (8, 9). However, it has been shown in several intervention studies (10–12) that these links are not an effect of dietary sugars as such but due to excess energy consumption. It has been scientifically proven that energy balance (equilibrium between energy intake and energy expenditure) is most relevant for body weight regulation, whereas single nutrients such as sugar, different macronutrient compositions or timing of the diet are more or less negligible (10, 13, 14). Keeping body weight within a healthy range is important, because overweight and obesity are the key drivers of various NCDs (15–17). Therefore, the primary goal in tackling overweight and obesity should be to reduce the individual’s energy intake. One approach is the reformulation of foods and beverages by decreasing their energy content (18). It has been demonstrated that lowering the energy density of foods can substantially reduce daily energy intake and could therefore be a powerful public health measure for weight management (19, 20). However, in recent years taste modulation has come into focus as a possible approach for influencing dietary behavior. In connection with the development of a “sweet tooth,” it is assumed that a reduction in the sweetness in foods and beverages might be helpful to prevent higher energy intakes and body weight gain (4, 6, 7). The idea is to modify sweetness preference by using less sweet foods and to attenuate consumers’ intrinsic desire for these foods, thus raising acceptance of a lower level of sweetness in their diet (21).

Therefore, the aim of this review is to elucidate the evidence for the question as to whether sweetness preference can by modulated by dietary sweetness and intake of sweet foods and beverages during an individual’s lifespan and whether this modulation affects energy intake and body weight.

## 2. Sweetness preference – childhood

In evolutionary terms, the preference for sweetness is innate in humans. Newborns react to the taste of sucrose with a pleasurable, laughing facial expression, whereas bitter or sour tastes produce a clearly aversive facial expression (2). There is broad discussion as to whether innate sweetness preference can be modulated by a generally sweet diet. If so, can such modulation occur in the first few days of life or already through the mother’s consumption habits *in utero*? A systematic review by Nehring et al. (22) addressed exactly this question by evaluating data from both intervention and observational studies.

The systematic review included studies that investigated whether the sweetness of the mothers’ diet before birth or in the first 6 months of infancy influenced the newborns’ taste and food acceptance later. The evaluation of both observational and intervention studies indicated great heterogeneity. Of 13 studies, six showed a statistically significant increase in sweet food intake and seven studies showed no difference in sweet food intake after exposure to sweet taste. Based on the analysis of intervention studies alone, seven out of 10 studies showed no effect on food intake and three studies showed increased food intake after exposure to sweet taste. Since the majority of the intervention studies did not find any effect of dietary sweetness on innate sweetness preference, the authors conclude that current data is too heterogenic for an appropriate conclusion to be drawn. Moreover, the authors point out that sweet tastes are naturally preferred, something which could limit further programming of sweetness acceptance (22). Subsequent literature research confirmed these findings by Nehring et al. (22), pointing out that data on early childhood exposure to sweet taste on sweetness preference was classified as inconsistent (1). Additionally, the European Society for Pediatric Gastroenterology, Hepatology and Nutrition (ESPHAN), which referred mainly to the findings of the systematic review of Nehring et al., also concluded that there is no causality between the consumption of sweet foods and sweetness preference during childhood (23). One of the main problems is that results from observational studies that suggest possible modulation of innate sweetness preference during early childhood can particularly be biased by a wide variety of other factors. A good example is children’s familiarity with a particular sweet drink. Increased consumption of a familiar sweet drink can diminish as soon as children become unfamiliar with the medium in which the sweet drink is served (24). Additionally, there is evidence that children’s preference for sweet foods increases significantly when their parents are particularly restrictive in their use of sweet foods (25). Therefore, results from observational studies must be viewed with caution.

Taken together, current scientific evidence does not permit the conclusion to be drawn that innate sweetness preference can be modulated by dietary sweetness during childhood. The high heterogeneity of the results from observational studies as well as interventional studies particularly indicate that additional research is needed in this area.

## 3. Sweetness preference – adulthood

In addition to the question of whether sweetness preference can be modulated during childhood, this question is also of great interest in adulthood. Although it is known that sweetness preference in adults is generally reduced compared to children (1), it could probably still be modulated by dietary sweetness, sweet foods or beverages. In a systematic review, Appleton et al. (4) evaluated diverse studies, which investigated sweet taste modulation in adults.

Various intervention studies examined high exposure to sweet foods versus low or no exposure to sweet foods in adults. Results from short-term intervention studies (duration of less than 1 month) indicate that the preference for sweetness decreased in adults after high exposure to sweet foods compared to control groups (4). For example, Griffioen-Roose et al. showed that a consumption of a predominantly sweet tasting diet for 24 h tended to result in an increased preference for savory snacks on the following day and lower preference for sweet foods (4, 26). In long-term studies (duration of three to 6 months), a reduction of dietary sweetness or a reduced intake of certain sweet foods did not result in reduced sweetness preference. Most of the intervention studies included attempted to reduce sweet foods, dietary sugars or other sweeteners in the diet to investigate the effect on taste preferences (4). For example, Wise et al. (27) examined in a randomized control trial whether participants who were fed a low-sugar diet for 3 months, in which 40% of energy from sugars was replaced with other non-sweetening macronutrients, had an effect on sweet taste and sweetness preference. Compared to the control group, which did not change their diet, the intervention group showed no difference in sweetness preference. Although the sweet taste of a vanilla pudding or a raspberry drink was perceived as sweeter, there were no differences between the control and intervention group in their preference for various sweetness levels in these foods. Other long-term studies showed comparable results, but mostly related to the reduction of a single sweet food or beverage. For example, the reduction of SSBs had no effect on the subsequent consumption of dietary sugars or other sweetened foods (4). However, since the publication of the systematic review by Appleton et al. (4), different studies that examined the long-term effects of sweet foods on sweetness preference have been published. Very recently, the study by Edwin Thanarajah et al. (28) examined whether subjects who were asked to eat a pudding with a high fat and sucrose content in addition to their diet for 8 weeks had any changes in their sweet or fat preference. Interestingly, the fat preference of the subjects for a low fat and sucrose pudding decreased compared to the control group after 8 weeks, but sweetness preference for that pudding was not affected. However, in view of previous results showing that sustained intake of sweet foods does not change sweetness preference, these results must be viewed with caution. The authors indicated several limitations, including that the effects observed might only be attributable to the pudding, meaning that the results are not transferable to other sweet foods, let alone fat and sugar as ingredients on their own. Furthermore, the authors did not control dietary intake during the whole intervention period, meaning that changes in dietary patterns due to the interventions themselves which might affect the results cannot be excluded (28).

All in all, current scientific evidence generally does not point to any modulation of sweetness preference in adulthood (4). However, it should be mentioned that some results from short-term studies tend to point in an opposite direction of sweetness exposure and sweetness preference [increased intake = decreased preference (26)].

## 4. Sweetness preference – energy intake and body weight

Besides the effects of modulation of dietary sweetness on sweetness preference only, there were also investigations focusing on the modulation of dietary sweetness on energy intake and body weight.

To investigate the effects of dietary sweetness on energy intake and body weight, results from intervention studies are mainly needed. Higgins et al. (7) recently evaluated the existing scientific literature on this issue. A systematic analysis of the literature showed that only seven of 804 studies were actually designed to investigate the effect of dietary sweetness on energy intake and body weight. Among the seven studies, only two were intervention studies. Therefore, data from intervention studies that looked at the effect of changing the overall dietary sweetness on energy intake and body weight are still scarce today.

Of these two intervention studies, the first one was a randomized control trial with a longer investigation period of 24 weeks. This trial investigated the effect of reduced dietary sweetness on energy intake and body weight gain. Sweetness reduction was achieved by a sugar-reduced diet through the reduction of sweet foods, including SSBs or sweetened cereals as well as fruit such as apples, mangos, raisins and others to achieve a reduction in sweetness compared to the control group, which maintained its normal diet and was not advised to eliminate sweet products from the diet. However, after an intervention period of 24 weeks, no differences in energy intake or body weight were detected between the intervention and control group (29). The second study was a cross-over controlled trial with a much shorter investigation period of 24 h. This trial investigated whether the consumption of a predominantly sweet tasting diet or savory tasting diet or a mixed diet for 24 h resulted in different *ad libitum* energy intake on the following day. Diets were matched for energy content and macronutrient composition. On the next day, no differences in energy intake were observed on an *ad libitum* lunch buffet (26). These findings suggest that neither the fairly long-term nor a very short-term modulation of dietary sweetness affects energy intake or body weight. However, these two intervention studies have considerable limitations, including a very short duration of exposure (24 h) and a small number of participants (39 subjects) (26), which is too short to indicate any possible long-term impacts, whereas the other study had a longer duration of 6 months but poorly controlled changes of dietary sweetness, because the participants were merely advised (but not continuously controlled) to reduce sweet foods and beverages (29). Consequently, there is a strong need for additional research and future studies with an adequate duration and participant numbers, which are well controlled and described for exposure (e.g., modulation of sweet foods and beverages, including the level of sweetness as well as frequency of intake). Furthermore, a standardized method to determine total dietary sweetness would improve the compatibility of single studies (7). In line with the findings of dietary sweetness, none of the other intervention studies that focused on the modulation of intake of certain sweet foods affected energy intake (4). For example, the consumption of sweetened breakfast cereals had no effect on energy intake compared to unsweetened ones over a survey period of 5 days (30) and the additional consumption of three different tasting high-energy snacks (hazelnuts, chocolate, or potato chips) over 12 weeks showed no differences in energy intake between these groups after the 12 weeks, respectively (31).

Taken together, there is currently no evidence that the modulation of dietary sweetness or certain sweet foods change energy intake or body weight. However, it should be mentioned that current evidence is scarce and limited, pointing to the need for additional research and future studies, with an adequate duration and number of participants as well as well-controlled sweetness exposure.

## 5. Conclusion

This review summarized current scientific literature, investigating the effect of dietary sweetness modulation as well as modulated intakes of sweet foods and beverages on sweetness preference, energy intake and body weight. The majority of the studies summarized did not show any impact from altering dietary sweetness on these endpoints. However, with respect to sweetness preference only, some studies indicate contrary preferences to dietary sweetness exposure in short-term.

For children, the findings of this review regarding sweetness preference are mainly based on the systematic assessment of Nehring et al. (22), which highlighted the great inconsistency between observational and intervention studies, focusing on sweetness preference modulation in childhood. These inconsistent findings were subsequently confirmed by the evaluation of Venditti et al. (1) in their scoping review. Contrary to findings from intervention studies, some results from observational studies showed possible associations between increased consumption of sweet foods and increased exposure to sweetness (22). At this stage, it must be pointed out that observational studies can only show correlations (associations) and not causality (cause and effect). Therefore, the scientific evidence is rated lower in the hierarchy of evidence compared to intervention studies (32). The main problem of observational studies is the difficulty in isolating the effect of one dietary or lifestyle factor from all the other factors that can influence taste preferences such as the parents’ diets and lifestyle, children’s familiarity with foods or certain tastes, and parents’ control of children’s diets, including prohibitions (33).

For adults, neither a modulation of overall dietary sweetness nor sweet foods had any effect on sweetness preference. This was especially the case in long-term studies, which showed equivocal results (4). However, it should be mentioned that some results in short-term studies point to contrary preferences with regard to dietary sweet exposure [increased exposure = decreased preference (26)]. In the short term, however, these findings contradict the theory of the development of a “sweet tooth.” In line with this, none of the studies in the systematic review by Appleton et al. or subsequent analyzes were able to confirm that heightened exposure to dietary sweetness or certain sweet foods increased the internal desire for more sweet foods (4, 28).

Currently, there are only two intervention studies that investigated the effect of changing the dietary sweetness on energy intake and body weight (26, 29). All other intervention studies focused on changes in sweet foods or beverages only (4). None of these studies showed any differences in energy intake and body weight gain after modulation of general dietary sweetness or certain sweet foods, which is certainly also due to the fact that none these studies modified dietary sweetness together with energy intake through the replacement with low- or no-calorie sweeteners (4, 26, 29). Further studies that will investigate a change of dietary sweetness on energy intake, body weight and other physiological parameters have already been announced (6). Based on the findings of the intervention studies, there is currently no scientific evidence that dietary sweetness modulation alone or the modulation of certain sweet foods and beverages affect total energy intake and body weight.

The findings that modulation of dietary sweetness neither affects sweetness preference nor energy intake and body weight, suggest either that there is currently no evidence for the assumption that there is a learned sweetness preference due to higher sweet exposure in the diet or that in view of the lack of evidence for a learned sweetness preference, a reduction of dietary sweetness in foods and beverages cannot be seen as an appropriate approach for tackling overweight and obesity. However, in connection with food reformulation to tackle overweight and obesity, it is often proposed that it is sufficient to reduce the sweetness of foods and beverages (4, 7). Hypothetically, by reducing dietary sweetness, consumers have an attenuated intrinsic desire for these foods and beverages and, therefore, accept a lower level of sweetness in their diet (21). Although the energy density of foods is determined by its water and macronutrient content (34), sweet foods are generally assumed to be energy-dense (35, 36). This leads to the assumption that a reduced desire for sweetness results in lower energy intake and can reduce body weight (6, 35). Again, this analysis of current data regarding sweetness preference, energy intake or anthropometric measures does not support this hypothesis.

To prevent body weight gain, food reformulation should not focus on the reduction of sweetness or other tastes but on the energy content of foods (37). Energy balance is relevant for body weight regulation, as demonstrated by various systematic reviews and meta-analyzes with the highest scientific evidence (10, 13, 14, 38). In line with this, it is also not plausible from a purely sensory point of view to focus on just one taste. Sweetness is only one component that contributes to the palatability of foods, because sweetness is usually consumed together with other tastes, such as sourness (e.g., fruit, sweetened yogurt) or fattiness (e.g., pastries or cakes). It is only logical that these other sensory properties also influence the palatability of foods (7).

Taken together, there currently appears to be little evidence that the modulation of dietary sweetness affects sweetness preference, energy intake or body weight. These findings question the idea of reducing the sweetness of foods and beverages to prevent overweight and obesity.

## Author contributions

PP: Conceptualization, Writing – original draft, Writing – review & editing.
